# Social Sentiment Sensor in Twitter for Predicting Cyber-Attacks Using *ℓ*_1_ Regularization

**DOI:** 10.3390/s18051380

**Published:** 2018-04-29

**Authors:** Aldo Hernandez-Suarez, Gabriel Sanchez-Perez, Karina Toscano-Medina, Victor Martinez-Hernandez, Hector Perez-Meana, Jesus Olivares-Mercado, Victor Sanchez

**Affiliations:** 1 Instituto Politecnico Nacional, ESIME Culhuacan, Mexico City 04440, Mexico; ahernandezs1325@alumno.ipn.mx (A.H.-S.); gasanchezp@ipn.mx (G.S.-P.); likatome@gmail.com (K.T.-M.); vicman27df@hotmail.com (V.M.-H.); jolivares@ipn.mx (J.O.-M.); 2 Department of Computer Science, University of Warwick, Coventry CV4 7AL, UK; v.f.Sanchez-Silva@warwick.ac.uk

**Keywords:** security, social sentiment sensor, hackers, social media, statistics, *ℓ*_1_ regression, Twitter, cyber-attacks

## Abstract

In recent years, online social media information has been the subject of study in several data science fields due to its impact on users as a communication and expression channel. Data gathered from online platforms such as Twitter has the potential to facilitate research over social phenomena based on sentiment analysis, which usually employs Natural Language Processing and Machine Learning techniques to interpret sentimental tendencies related to users’ opinions and make predictions about real events. Cyber-attacks are not isolated from opinion subjectivity on online social networks. Various security attacks are performed by hacker activists motivated by reactions from polemic social events. In this paper, a methodology for tracking social data that can trigger cyber-attacks is developed. Our main contribution lies in the monthly prediction of tweets with content related to security attacks and the incidents detected based on $\ell_{1}$ regularization.

## 1. Introduction

Online Social Networks (OSNs) are platforms designed as communication channels for information exchange in real time. These platforms may generate over 1 billon posts per month around the world. For example, Twitter statistics [1,2] report the generation of 313 million posts monthly, better known as tweets, over different countries.

Different topics in Twitter may reflect polarized opinions from celebrities, corporations, and regular users about daily life aspects [3], some of them with well defined geographic embedded data (e.g., assisted GPS coordinates). Streams of tweets generate valuable information that can be modeled as a social sentiment sensor for real-world event prediction [4] by analyzing clustered topics, such as in rumour spreading analysis [5], human mobility sensing [6], spam & botnet detection [7], and disaster response [8].

Within the context of cyber-security, the large volumes of data that can be collected over different time intervals from Twitter have the potential to facilitate the understanding of the motivation behind cyber-attacks by sentiment analysis of tweets. Specifically, any underlying correlation among the sentimental polarity of various groups of Twitter users can be interpreted by probabilistic and classification models [9], whose results are predictive by nature and can be used as a social behavior warning tool. For example, in [10], an early warning process related to abnormal behavior is developed relating intrusion techniques and terrorist attacks.

Regional language and lexical variations derived from tweets are key factors in searching patterns related to sentimental tendencies. Natural language processing has shown that negative-oriented textual features [11] related to information security lexicons used by hacktivists groups can be used as warning alarms to mitigate possible cyber-attacks. Therefore, important political, religious, and cultural events can serve as targets for data extraction in Twitter to predict such attacks [12].

This paper focuses on sentiment analysis extracted from tweets, which are processed with probabilistic techniques [13] in order to measure the correlation between the sentiment of user groups and possible cyber-attacks. Specifically, we propose a methodology for predicting possible cyber-attacks based on scraping and classifying Twitter data. This is done by employing a supervised learning algorithm [14,15,16,17] on a daily corpora of tweets. The methodology classifies tweets in order to obtain monthly sentimental scores that are fitted into an $\ell_{1}$ regression algorithm to predict potential cyber-attacks.

Although current advances on information security have improved the trust on information handling mechanisms by means of e.g., cryptographic and data protection algorithms [18,19,20], cyber-attacks are still an important issue to tackle. Our methodology has the potential to aide in the prevention of cyber-attacks based on sentiment analysis of tweets.

The rest of the paper is organized as follows. Section 2 provides a review of the related work. Section 3 describes the proposed methodology. Section 4 describes the data gathering and pre-processing mechanisms used. Section 5, Section 6 and Section 7 detail the supervised classification used in this work. Section 8 describes the statistical analysis. Section 9 provides the experimental results. Finally, Section 10 concludes this work.

## 2. Related Work

According to [21], cyber-attacks are increasing as a result of global insurgency given geopolitical contexts. These attacks pose major concerns due to their potential effects on denial-of-service, data leaking, and application compromising. Alternative security measures, like forecasting threatening security events, are thus gaining credibility.

Data from OSNs are useful for extending capabilities from intrusion detection systems (IDSs) and intrusion prevention systems (IPSs) from outer-level networks. In [22], a Latent Dirichlet Allocation (LDA)-based model is proposed to discover semantically related concepts to analyze cyber-crime forensics. More recently, a bipartite and monopartite network analysis is achieved by crawling hackers forums to identify members by specific malicious tool usage [23]. A list of anti-threat strategies is proposed in [24] to prevent and visualize common practices regarding privacy, spamming and malicious attacks. In [25], the authors present a relationship of social unrest between countries and directed cyber-attacks. These works prove that Arbor Network data are useful to determine if attacks such as Distributed Denial-of-Service (DDoS) attacks are expected to grow if radical or extremist sentiments from users are perceived in streams of OSN posts.

Predictive analysis is particularly advantageous in Twitter due to the fact that certain functionalities, such as retweets, favorites, and replies, can be characterized and, together with the polarity of the text, can provide data that increase the forecasting of events such as political elections and product outcomes [26]. According to [27], the predictive power of social networks can be exploited by the inspection of published data and statistical modelling, which may result in the detection of a statistical relationship between a social media-based measure (e.g., number of re-tweets or sentiment analysis scores) and the outcome of interest (e.g., economic growth or presidential approval rates). For example, in [28], an $\ell_{1}$ regularized regression model is presented in order to predict influenza-like illness by training data from Twitter and comparing outcomes with official health reports.

## 3. Proposed Methodology

**Figure 1:**
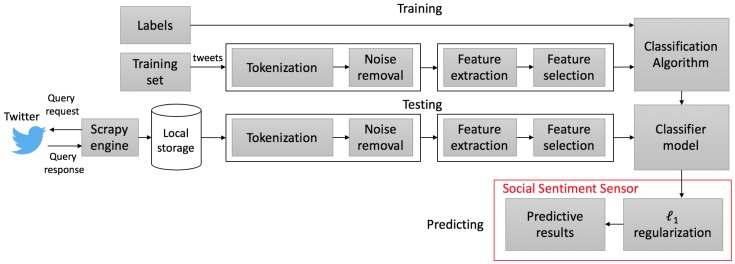
Work flow of the proposed methodology.

The work flow of the proposed methodology is depicted in Figure 1. First, a query is requested from the Twitter search endpoint. The resulting response containing blocks of tweets is then processed by a web scrapping engine and stored on a local database. A set of pre-selected tweets is prepared for training a classifier using supervised learning [14,15,16,17]. Finally, sentimental scores of the classified tweets are fed to an $\ell_{1}$ regularization algorithm to obtain predictive results.

## 4. Data Gathering and Pre-Processing

### 4.1. Data Acquisition

**Figure 2:**
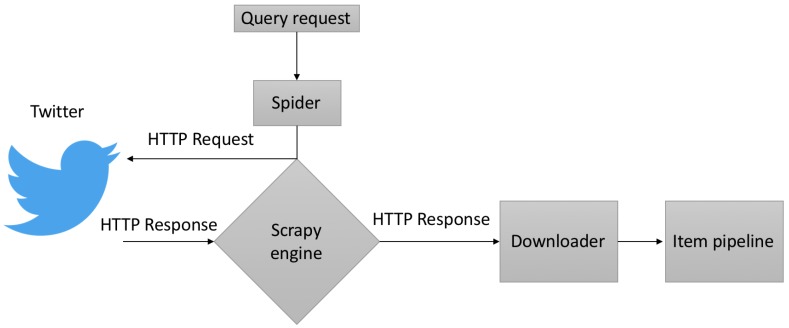
Data gathering scheme.

Data gathering schemes are designed for querying Twitter endpoints to obtain chronological tweets. Recent works on sentiment analysis [29,30,31] use a public information streaming platform known as Twitter Standard Search API, which is an interface that has capabilities for information retrieval in chronological order for no longer than seven days [32]. In this paper, we use an approach proposed in [33] for historical retrieval by querying Twitter search endpoints. The web crawling tasks are done with web spiders’ engines designed for document scraping in an automated and efficient manner. Information is processed by Scrapy, a Python Web Scraping Framework that extracts embedded text in HTML tags and simultaneously uses recursive functions to analyze each link to follow other tweets. This data gathering scheme is depicted in Figure 2.

Collecting data is achieved by querying the endpoints in time intervals sorted by days. Each query *q* is based on *n-grams* (set of co-occurring words within a given text) bags-of-words related to specific events defined as *q* = [{*1-gram*, *2-gram*, *3-gram*, …, *n-gram*}, {*date*}].

**Figure 3:**
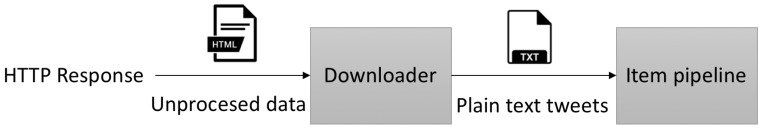
Embedded text in HTML.

Queries responses are processed by a web spider towards the endpoint and redirected to a Scrapy download layer. Unprocessed data are then fed into the Scrapy engine in order to strip hypertext tags and retrieve each tweet in plain text. As depicted in Figure 3, the retrieved text is processed independently in Scrapy pipes that handle data streams into objects to be stored on a relational database.

The set of retrieved queries is the corpus of tweets, C, and its size is directly proportional to the daily number of tweets stored for the query. Each tweet can be represented as a structure comprising fundamental attributes, as tabulated in Table 1.

Each tweet *t* is stored with its own id as a primary key that is used to sort them in a sequentially and non-repeatable way. Each tweet in set C is then denoted by $C\left(q\right)=t_{i}\in {\left\{t_{id},t_{text},t_{date}\right\}}_{i=1}^{n}$.

### 4.2. Tokenization and Noise Removal

A cleaning task is applied on C to generate individual arrays of words (i.e., tokens) for each tweet. A normalization step is required to transform each token into lower case words; a dimensionality reduction [34] of C is important to reduce textual noise. Noise is considered as frequent uni-grams or stop-words (very commonly used words) that do not provide valuable information as candidate textual markers. In the case of the English language, sets of *stop-words* widely applied in Natural Language Processing are used in text cleaning tasks. This work uses the publicly-available English stop-words set published in [35], and each word is weighted by textual and lexical functions in a sentence [36]. URL patterns are removed from the corpus. Other non-informative expressions, such as retweets *RT* and appearances of @username, are also deleted.

### 4.3. Lexical Derivations

Textual markers have lexical derivations as part of ungrammatical text structures written by most users. Grammatical restriction is performed to stem each token, thus avoiding repeated samples from the same grammatical root and bias in the training step for classification. An example of stemming is shown in Table 2.

We use a Snowball Stemmer for lexicographical lemmatisation, which is a set of probabilistic algorithms based on Porter stemmer [37] of Indo-European languages and has been shown to attain high capabilities for searching pattern inflections into roots from composed words [38].

## 5. Pre-Classification and Class Labeling

Supervised classification provides predefined class labels given specific inputs, where each class must be independent from the others. Selecting relevant and high impact tweets are important for good training performance due to the fact that some words give most information about a particular context. We use The Stanford sentiment corpus [39] along with tweets crawled by our own scraping approach, tweets are labeled as negative (neg) or positive (pos) based on the user’s emotions.

A second set of tweets related to cyber-security and cyber-attacks topics is scraped by querying terms contained in The Glossary of Common Cybersecurity Terminology [40], and other manually annotated hacker-activists terms [11]. It is important to mention that hacktivism, according to [41], is a type of activity among hackers with specific political motivations and ideologies, such as religion or jigonism. In general, there are four motivations, i.e., revenge, financial, notoriety and curiosity, [42] related to hacktivism. This work considers all of these motivations.

**Figure 4:**
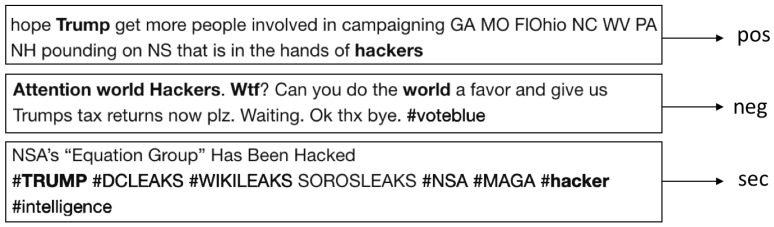
Example of labeling for the three observed classes.

Crawled tweets are labeled by a sec(security−oriented) tag. The set of labels is then denoted by label={pos,neg,sec} and the corpus for training is denoted by $T=\tau_{i}\in {\left\{tweet_{text}^{label}\right\}}_{i=1}^{n}$, where $\tau_{i}$ is the ith tweet text and label in the training set. Figure 4 depicts some examples regarding class labeling.

## 6. Supervised Classifier

**Figure 5:**
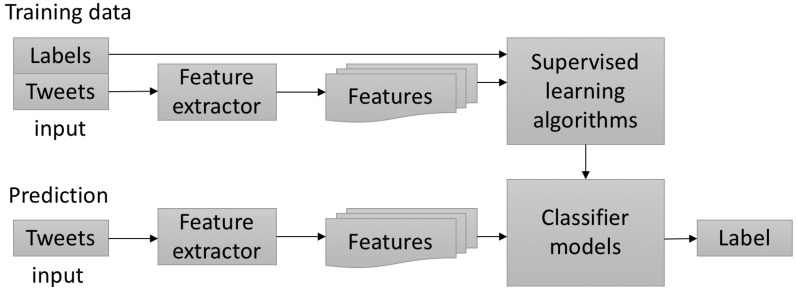
Training and label prediction.

Building a supervised classifier is achieved by first transforming each input of textual markers into features, followed by a training step with labels. Features extracted from *T* contain basic information that allows for C to be successfully classified. The work flow is graphically depicted in Figure 5.

Features and labels from *T* are processed by the supervised learning algorithms [14,15,16,17] to generate a classifier model. A feature extractor computes features based on words by the term frequency-inverse document frequency (*Tf-idf*) algorithm [43]. A label for each tweet of C is then predicted.

### Feature Extraction and Selection

Features are based on sentimental relevance; i.e., words that better describe a user’s sentiment towards a specific context are selected. As proposed in [44], identifying raw *n-grams* is more useful for feature extraction than using speech tagging because supervised classifiers tend to attain a higher accuracy with grammatical and positional independence in sentences.

In order to avoid over-fitting, we perform a model selection procedure to split data into random matrices for training and testing. By performing a *train–test* selection procedure with Python sklearn library, we divide *T* into 80% training and 20% validation subsets. Training and validation tweets from regular users merged with security oriented users are denoted by $X_{T}$, which contains pre-processed text from tweets, while *y* denotes their respective labels. Resulting subsets from *T* are denoted by $X_{T},y_{T}$, which are the training subset tuples, and $X_{V},y_{V}$, the validation subsets tuples selected to evaluate the classifier model. Word particles contained in tweets from the training set are extracted and transformed into *Tf-idf* term weights [45] by using a sklearn *Tf-idf* vectorizer; then, each resulting vector is normalized by an $\ell_{2}$ norm.

## 7. Classification Baseline

Choosing a good classifier is an important task to generate a robust model for testing corpus C. In other words, results must be accurate enough to eventually find relationships between the users sentiments and cyber-attacks. In [39,46,47], different classifiers such as Naive Bayes, Maximum Entropy and Support Vector Machines are proposed and evaluated; results show that, for noisy labels and the case of emotions in tweets, Support Vector Machines attain better results than those of other text classifiers.

### 7.1. Naive Bayes Classifier

Classifiers based on the Bayes theorem are widely used in text classification [14] for short messages like tweets because of the simplicity in computing probabilistic evidence for class prediction given independent text features. This method contrasts with those that employ Bernoulli models [48], which are based on document counts for each class. Having a label set for *C* classes, we can define parameters to calculate the probability of a class *c* given a tweet by:

(1) $$P_{NB}\left(c|t\right)=\frac{\left(P\left(c\right)\right)\sum_{i=1}^{m}p{\left(f_{i}|c\right)}^{n_{i(t)}}}{P(t),}$$

where *t* is a tweet, *c* a class (label), $f_{i}\in f\left(X_{T}\right)$ is the feature, and $n_{i(t)}$ is a word presence given *t* and *m* is the number of features.

### 7.2. Support Vector Machines

Support Vector Machines [15] are suitable for bounding data in linear and non-linear ways. Inherently, SVM is a binary classifier, meaning that data are separated into two labeled classes. For a multi-class approach for the training set $\left(X_{T},y_{T}\right)$ with labels $y_{T}\in \left\{0,1,2\right\},$ an optimization approach is proposed by solving:

(2) $$\phi \left(w,\xi \right)=\frac{1}{2}{∥w∥}^{2}+C\sum_{i=1}^{\ell }\sum_{m\neq y_{i}}\xi_{I}^{m}.$$

Constrained to $\left(w_{y}\cdot t_{i}\right)+b_{y_{i}}\geq \left(w_{m}\cdot t_{i}\right)+b_{m}+2-\xi_{i}^{m}$, $\xi_{i}^{m}\geq 0,i=1,\ldots ,\ell ,m\in \left\{1,\ldots ,k\right\}$, thus we can find an optimized decision function by finding the saddle point of the Lagrangian:

(3) $$f\left(x,\alpha \right)=\arg\max_{n}\left[\sum_{i=1}^{\ell }\left(c_{i}^{n}A_{i}-\alpha_{i}^{n}\right)\left(t_{i}\cdot t\right)+b_{n}\right],$$

where *w* is the hyper plane, $\alpha_{i}$ is the non-negative Variable Lagrange Multiplier, $y_{i}$ is the *i*th input class (label) from the label set, *t* are input tweets, *b* denotes the hyper-plane parameters (bias), ξ is a slack variable (0<ξ≤i is the point between the margin and the correct side of the hyper-plane with ξ>1 denoting a misclassified point) and *C* is the regularization parameter.

### 7.3. Maximum Entropy Classifier

Maximum Entropy classifiers are widely used for learning from input features in a weighted manner to generate a discriminative model that evaluates possible values from possible classes [16,17]. The model is represented by:

(4) $$P_{ME}\left(c|t\right)=\frac{1}{Z(t)}exp\left(\sum_{i=1}^{n}\lambda_{i,c}F_{i,c}\left(t,c\right)\right),$$

where *c* denotes the class (label), *t* is a tweet, λ is the weight vector (considering that a higher weight assumes a strong indicator about the class), Z(t) is the normalization function given *t*, and $F_{i,c}$ is the feature-class function for a feature $f_{i}\in f\left(X_{T}\right)$.

## 8. Prediction—Statistical Analysis

### $\ell_{1}$ Regularized Regression

Regression is suitable for predicting events given multiple inputs, better known as observations, that are linearly independent from each other [49]. A linear model is interpreted as:

(5) $$f\left(X_{C}\right)={\hat{y_{C}}}_{security\_oriented}=\beta_{0}+\beta_{1}X_{C_{pos}}+\beta_{2}X_{C_{neg}}+\varepsilon ,$$

where:

1. $X_{C}$ is the observation matrix of all classified tweets from corpus C
2. $X_{C_{pos}}$ and $X_{C_{neg}}$ are the observations with positive and negative scores, respectively,
  (a) $X_{C_{pos}}=\sum_{i=1}^{n}C_{i}\left(positive\right),$
  (b) $X_{C_{neg}}=\sum_{i=1}^{n}C_{i}\left(negative\right).$
3. ${\hat{y_{C}}}_{security\_oriented}$ is the fitted security-oriented response from regression coefficients $\left[\beta_{1},\beta_{2}\right]$ extracted from $y_{C_{security\_oriented}}=\sum_{i=1}^{n}C_{i}\left(security\_oriented\right)$.

Because of the negative effect on computing regression by ordinary least squares over highly correlated observations and an increase of variance, a regularized regression using selection and reduction is proposed. Regression based on vector norm $\ell_{1}$ can adjust the linear model by making some coefficients zero, which is suitable for large multivariate observation matrices. LASSO (Least Absolute Shrinkage and Selection Operator) is an adaptation to linear models that minimizes the error in the limit of absolute values from prediction coefficients:

(6) $${\hat{\beta }}^{lasso}=\arg\min_{\beta \in R^{P}}∥X_{C}\beta -y_{C}{∥}_{2}^{2}+\lambda {∥\beta ∥}_{\ell_{1}},$$

where λ is the tuning parameter for shrinking coefficients [β]. To solve the $\ell_{1}$ penalization problem, the Forward Stagewise algorithm [50] is computed. The proposed solution of Equation (6) is given by tacking the subgradient:

(7) $$\sum_{i=1}\left(y_{C_{i}}-X_{C_{i}}\beta \right)\left(-X_{C_{i,j}}\right)+\lambda g_{j},$$

where $g_{j}$ is the subbgradient of the $\ell_{1}$ norm, $g_{j}=sign\left(\beta_{j}\right)\;\mathrm{if}\;\beta_{j}\neq 0,g_{j}\in \left[-1,1\right],\;\mathrm{otherwise}$. The algorithms for the proposed system are shown in Algorithms 1–4.

**Algorithm 1:** Training Samples

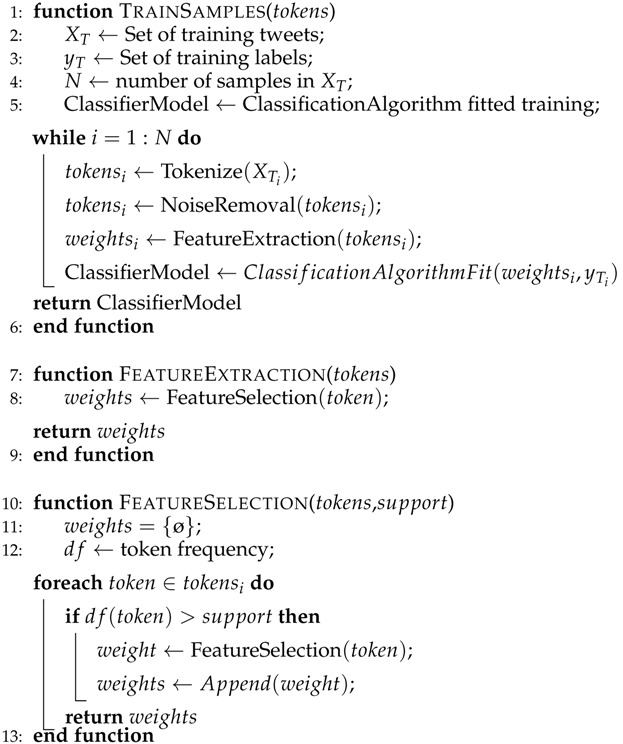



**Algorithm 2:** Testing Samples

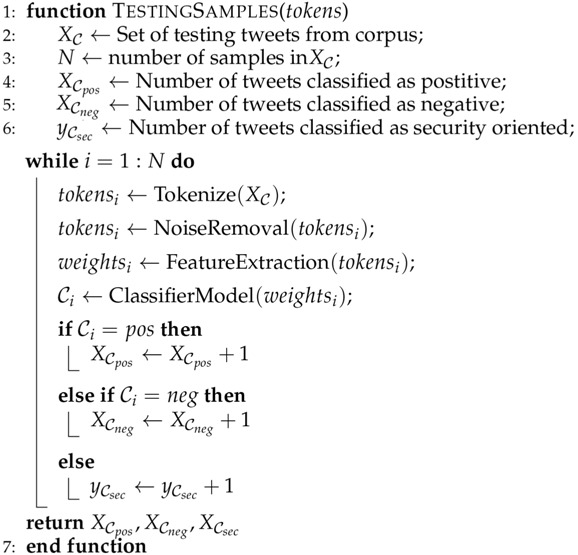



**Algorithm 3:** Computing the *ℓ*1 solution by Forward Stagewise

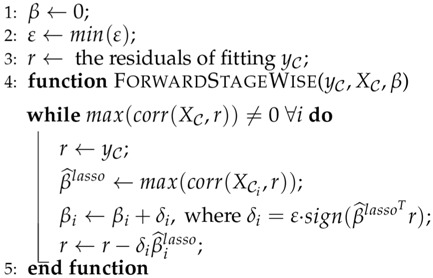



**Algorithm 4:** Predictions with regularized coefficients1: **function** Predict($X_{C_{neg}},X_{C_{pos},\beta_{i}}$)2:  *β* ← [*β*_0_, *β*_1_, *β*_2_];3:  ${\hat{y_{C}}}_{security\_oriented}\leftarrow \beta_{0}+\beta_{1}X_{C_{pos}}+\beta_{2}X_{C_{neg}}+\varepsilon$; **return**
${\hat{y_{C}}}_{security\_oriented}$4: **end function**

## 9. Experimental Results

This section shows the evaluation of the proposed sensor for sentiment analysis using a total of 1,800,000 tweets in English. One million tweets are extracted using the method proposed in [33] from regular and cyber-security related accounts and 800,000 belonging to the Stanford dataset [51]. In Table 3, some well identified Twitter accounts related to *hacktivists*, cyber-security feeds, researchers, and enthusiasts users are tabulated.

Table 4 tabulates the classification results attained by the Support Vector Machine (SVM), Naive Bayes (NB), and Maximum Entropy (ME) classifiers. Bold rows represent the best classification results. These results are obtained using parameters related to document frequency (*df*), which is a threshold for support applied to weight terms where the minimum and maximum support are in the interval [0.5, 0.95].

### 9.1. A Case Study

During the 2016 United States of America (USA) presidential campaigns and post election time, an important set of polarized opinions was generated by Donald Trump polemic speeches. Speculations about the winning candidate increased by adding financial, political, immigration, religious, and sexist comments towards his opponent, Hillary Clinton, during the campaign. *Hacktivists* generated public threats towards Donald Trump using hash-tags like #OpTrump and #OpDrumpf. In addition, rumors about hackers manipulating electoral campaigns increased users’ negative reactions towards both candidates. Table 5 tabulates classification results of the three classifiers used in this work for a number of tweets generated by users who tweet regularly and *hacktivists*. These tweets are contained in C. As specified before, we denote by $X_{C}$ the testing set to perform this case study. In order to better appreciate the sentimental average scores, i.e., $X_{C_{neg}}$, $X_{C_{pos}}$ and $y_{C_{security\_oriented}}$, the 486 tracked days between 9 January 2016 and 1 May 2017 are divided into six time-intervals.

### 9.2. Regularized Regression

Prediction over high volumes of scores can be difficult with ordinary regression due to unbiassed coefficients. By employing LASSO [52], we can shrink coefficients in order to optimize our prediction model. Moreover, regularized regression tasks can be only implemented in multivariate sets. As tabulated in Table 4, the ME classifier attains the best accuracy results, so we use $\ell_{1}$ normalization on ME [53]. We divide the normalized scores from $X_{C}$ into monthly prediction tasks to precisely analyse the presidential campaign period. A statistical report containing the following measures is tabulated in Table 6:

- Mean Squared Error (M.S.E.): shows the difference or loss of the predicted scores with the inputs, i.e., between the actual scores, $y_{C_{security\_oriented}}$, and the predictions, ${\hat{y_{C}}}_{security\_oriented}$.
- *p*-value (probability value): determines how well the observations ($X_{C_{neg}}$, $X_{C_{pos}}$) are adjusted in the predictive model, thus rejecting the null hypothesis that relates to the low effectiveness of the samples. The lower the probability value (*p*-value ≈ 0), the greater the adjustment in the model.
- $R^{2}$ (coefficient of determination): explains the proportion of adjustment from the observations, ($X_{C_{neg}}$, $X_{C_{pos}}$), with respect to the outputs, ${\hat{y_{C}}}_{security\_oriented}$.
- Detected Attacks : the total number of cyber-attacks detected.

**Figure 6:**
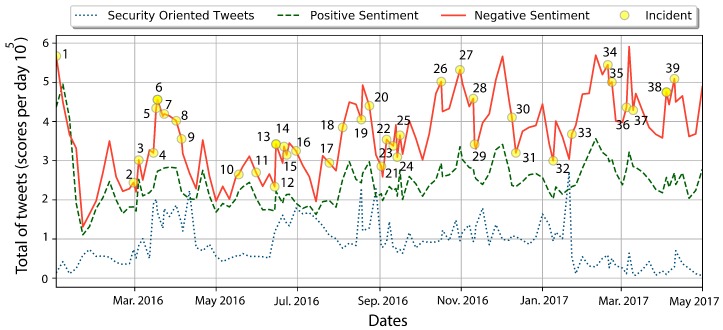
Chronological user’s sentiments and reported security-related incidents.

**Figure 7:**
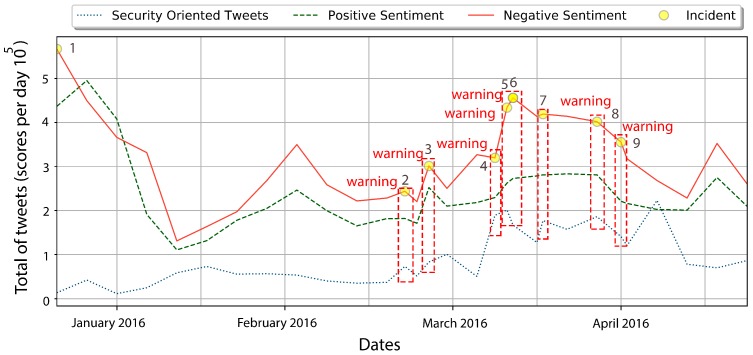
Proposed proof of concept.

Bold rows represent the maximum correlation between users sentiment and a security oriented response given by $R^{2}$. Historical data extracted from Google News can help to determine if $R^{2}$ values related to users’ sentiments are correlated with cyber-attacks. During mid-March 2016, Trump’s comments and behavior regarding abortion, the violence on his rallies, and his declarations about the Brussels terrorist attacks, increase users’ negative opinions towards him and, in retaliation, hacktivists started a raid under the banner of OpTrump threatening election sites, voice-mails, and public information. June 2016 was also a hard month during the election; rumors about hackers hijacking elections by cyber-intrusions increase people’s reaction by posting DNC compromised servers revealing Hillary Clinton’s private emails. The observations obtained in these time series show that there is a correlation between the negative opinions expressed in tweets of hacktivists and cyber-attacks. A chronological time-line of tweets classified by ME as negative, positive, and security-oriented, as well as the index of important security related incidents, as reported by Google News (see Table 7), is presented in Figure 6. Based on the results, note that it is possible to define thresholds for predicting possible cyber-attacks, for example when the coefficient of determination, $R^{2}$, increases above 80%. Figure 7 depicts a PoC (Proof of Concept) of this idea. Specifically, this figure depicts the cyber-attacks perpetrated from January to April 2016 when the coefficient of determination, $R^{2}$, is greater than or equal to 80%. At the end of February 2016, the hacktivist Anonymous made the following statement: *Suspend campaign…or face consequences*. Anonymous also created sites like www.optrump2016.com (now redirected to www.donaldjdrumpf.com) with a counter for the time left before hacking sites related to the presidential campaign. Simultaneously, the number of tweets with an associated sentiment increased considerably from February to March, 2016, approximately 78% for negative tweets, 87% for positives tweets, and 37% for security-related tweets. This confirms that the correlation computed by Equation (6) is useful to predict possible cyber-attacks.

## 10. Conclusions

This paper presented a methodology to predict cyber-attacks by using a Social Sentiment Sensor in Twitter. The methodology collects historical tweets and classifies them as negative, positive and security-oriented. By using $\ell_{1}$ regularization on the classified tweets, cyber-attacks can be predicted when the corresponding coefficient of determination reaches a certain value. The methodology is evaluated within the context of the 2016 USA presidential campaigns, during which politicians appear to have influenced the sentiment of Tweeter users and in response, hacktivists reacted as part of the opposition by threatening public information. Specifically, we have shown that the proposed methodology can serve as a warning mechanism to detect possible cyber-attacks.

The proposed methodology is not limited to cyber-attacks. Our future work includes testing and tailoring the proposed methodology to predict other real-life events such as pandemics, political alignment, and market events.

## Figures and Tables

**Table 1:** Tweet object.

Attribute	Description
id	the integer representation of the unique identifier for this Tweet
created_at	UTC time when a tweet was created
text	The actual UTF-8 text of the status update

**Table 2:** Stemmed lexical variations.

Prefix	Root	Sufix
none	corrput	tion
none	corrupt	ed
none	incorrupt	ibility

**Table 3:** Identified Twitter accounts related to hacking and cyber-security.

Account Type	Identified Accounts
hacktivism	anonymouspress, youranonglobal, wapoanon, werallanonymous, observingsentin, theanonmovement, freeanons, global_hackers, anonymousvideo, anonrrd
cyber-security feeds and sensors	nitdefender, malwarebytes, oinionid, moixec, uscert_gov, nakedsecurity, kaspersky, fsecure, nortononline, nsc
researchers and enthusiasts	peerlyst, cyber, mikko, briankrebs, nieljrubenking, dangoodin001, gcluley, campuscodi, peterkruse, e_kaspersky, troyhunt, swiftonsecurity, icheylus

**Table 4:** Classification results of NB, SVM and ME.

Classifier	Class	Precision	Recall	$F_{1}$ Score
NB	negative	0.77	0.80	0.79
	positive	0.76	0.76	0.76
	security-oriented	0.94	0.91	0.93
SVM	negative	0.80	0.80	0.80
	positive	0.78	0.80	0.79
	security-oriented	0.95	0.94	0.95
ME	negative	**0.81**	**0.80**	**0.80**
	positive	**0.78**	**0.80**	**0.79**
	security-oriented	**0.96**	**0.94**	**0.95**

**Table 5:** Classified tweets over 486 days.

Dates	Classifier	pos	neg	sec
9 January 2016 to 23 March 2016	NB	1,858,329	2,143,213	535,449
	ME	26,451,360	2,920,311	450,793
	SVM	2,792,088	2,346,357	540,059
24 March 2016 to 12 June 2016	NB	1,909,028	1,969,211	1,969,211
	ME	24,294,780	2,384,148	569,337
	SVM	2,564,449	2,347,377	682,077
13 June 2016 to 1 September 2016	NB	1,957,351	2,428,557	1,208,306
	ME	24,017,220	27,840,39	1,013,131
	SVM	2,535,151	2,740,485	1,213,509
2 September 2016 to 21 November 2016	NB	2,290,596	2,966,951	951,907
	ME	28,019,700	3,308,982	802,142
	SVM	2,957,635	3,257,319	961,466
22 November 2016 to 10 February 2017	NB	2,456,003	3,217,832	985,666
	ME	30,309,120	3,480,291	827,089
	SVM	3,199,296	3,420,468	923,691
11 February 2017 to 1 May 2017	NB	2,436,753	3,464,375	237,160
	ME	29,392,200	3,703,008	198,667
	SVM	3,102,510	3,626,100	238,128

**Table 6:** Regularized regression measures report.

Months	MSE	$\beta_{1}$	$\beta_{2}$	*p*-Value	$R^{2}$	${y_{C}}_{\mathrm{security}\_\mathrm{oriented}}$	${\hat{y_{C}}}_{\mathrm{security}\_\mathrm{oriented}}$	Detected Attacks
January (2016)	0.00243	1609.36	845.54	0.0	0.61	116,910	70,146	2
February (2016)	0.00223	1609.36	845.54	0.0	0.63	210,874	132,850	1
**March (2016)**	**0.00001**	**1609.36**	845.54	**0.0**	**0.81**	**317,625**	**257,276**	**6**
April (2016)	0.00314	1609.36	845.54	0.0	0.54	372,438	249,533	2
May (2016)	0.00141	1609.36	845.54	0.0	0.67	122,674	83,531	2
**June (2016)**	**0.00002**	**1609.36**	**845.54**	**0.0**	**0.89**	**223,674**	**199,069**	**6**
**July (2016)**	**0.00008**	**1609.36**	**845.54**	**0.0**	**0.86**	**230,655**	**198,363**	**1**
**August (2016)**	**0.00009**	**1609.36**	**845.54**	**0.0**	**0.85**	**410,874**	**349,242**	**3**
September (2016)	0.00015	1609.36	845.54	0.0	0.77	291,643	224,565	2
October (2016)	0.0004	1609.36	845.54	0.0	0.71	241,438	188,321	2
November (2016)	0.00054	1609.36	845.54	0.0	0.79	230,123	181,797	2
December (2016)	0.00312	1609.36	845.54	0.0	0.53	229,451	121,609	2
January (2017)	0.00144	1609.36	845.54	0.0	0.69	378,286	261,017	1
February (2017)	0.00334	1609.36	845.54	0.0	0.52	107,933	56,125	1
March (2017)	0.00339	1609.36	845.54	0.0	0.51	96,973	49,456	1
April (2017)	0.00330	1609.36	845.54	0.0	0.56	94,961	53,178	1

**Table 7:** News reporting security-related incidents.

Index	Date	News	Source	Negative Sample	Security-Oriented Sample
1	2 January 2016	’Anti-IS group’ claims BBC website attack	BBC News	56,712	1573
2	2 January 2016	Hackers Shut Down Donald Trump Election Campaign Website	Hack Read	56,712	1573
3	29 February 2016	US Cyber Command launches hacking offensive against Islamic State	Washington Times	24,378	5929
4	4 March 2016	Donald Trump’s voicemails hacked by Anonymous	The Independent	30,141	7744
5	15 March 2016	Anonymous Declares ‘Total War’ On Donald Trump With Cyber Attacks Planned For 1 April	Huffington Post UK	31,977	16,940
6	15 March 2016	Anonymous Just Declared War on Donald Trump With a Massive Cyberattack	MIC	31,977	16,940
7	17 March 2016	ANONYMOUS OPTRUMP: HACKERS LAUNCH ‘TOTAL WAR’ ON DONALD TRUMP IN REVENGE FOR ‘HATEFUL’ CAMPAIGN	The Independent	43,401	29,282
8	18 March 2016	Trump Under Attack: The Donald Is Hacked by Anonymous and Son Eric Receives Threatening Letter Containing White Powder	People Magazine	45,594	14,762
9	23 March 2016	Anti-Trump campaign sparks civil war among Anonymous hackers	The Guardian	41,922	8107
10	1 April 2016	Anonymous Will Begin Latest War on Donald Trump Friday, April Fools’ Day	Inverse	40,188	7623
11	5 April 2016	Donald Trump’s hotel chain HACKED for second time in six months	Mirror.co.uk	35,547	16,577
12	8 May 2016	Presidential candidates may be vulnerable to foreign hackers, US says	The Guardian	26,469	6534
13	31 May 2016	Hacked construction signs call Trump a ‘shape shifting lizard’	FOX 4 News	26,979	6538
14	14 June 2016	Russian Spies Hacked Into the DNC’s Donald Trump files	CNN	23,358	13,794
15	14 June 2016	Russian Gov Hacks DNC, Steal Trump Oppo	The Weekly Standard	23,358	13,794
16	15 June 2016	Donald Trump Lone Hacker Claim Responsability for Stealing Democratic Party’s Data	ABC	34,221	14,762
17	21 June 2016	Russian hackers reportedly access Clinton Foundation	The Sidney Morning Herald	33,609	17,908
18	23 June 2016	Russian Hackers Targeted Hillary Clinton Campaign Google Accounts	Forbes	31,467	16,456
19	30 June 2016	Hacker Reveals New Trove of DNC Documents and Answers a Few Personal Questions	Mother Jones	32,487	18,388
20	25 July 2016	FBI Suspects Russia Hacked DNC; U.S. Officials Say It Was to Elect Donald Trump	Daily Beast	29,427	12,826
21	4 August 2016	Hackers for Hillary: event attendance ‘through the roof’ after Trump remarks	The Guardian	38,505	8954
22	18 August 2016	Is Russia hacking the US election?	BBC News	40,494	9075
23	24 August 2016	No proof, but ‘Russian hackers’: CNN blunders with report on ‘breach’ at NYT–not even asking NYT	International RT	44,013	8833
24	2 September 2016	Putin on DNC hack: Let’s talk content, not hackers’ identity	International RT	28,560	9438
25	6 September 2016	Hillary Clinton Suggests Alleged Russian Hacking Is Designed to Help Trump	NBCNews.com	35,394	10,890
26	11 September 2016	CIA Director John Brennan warns of Russian hacking	NewsHour	33,762	9075
27	14 September 2016	Trump a ‘National Disgrace,’ Colin Powell Wrote in Hacked Emails	ABC News	36,465	7865
28	17 October 2016	Could Russian hackers change the U.S. election result?	Aljazeera	50,184	11,374
29	31 October 2016	Was a Trump Server Communicating With Russia?	Slate Magazine	53,193	11,253
30	10 November 2016	Russian hackers throw Trump victory party with new spear phishing campaign	Ars Technica	45,849	11,011
31	11 November 2016	Russia-linked DNC hackers launched wave of cyberattacks hours after Trump victory	Ars Technica	34,170	11,737
33	2 December 2016	Trump condemns CIA Russia hacking report	BBC News	31,977	12,463
32	9 December 2016	Russian Hackers Acted to Aid Trump in Election, U.S. Says	New York Times	41,055	12,705
34	9 January 2017	Surprise! WikiLeaks’ Assange Backs Trump on Russia Hacking Report	NY Times	36,771	11,132
35	22 February 2017	U.S. CyberCorps, ROTC For Hackers, In Disarray in Trump Admin	Vocativ	50,082	5929
36	5 March 2017	DeepStateGate: Democrats’ ‘Russian Hacking’ Conspiracy Theory Backfires	Big Government	43,605	13,331
37	10 March 2017	Trump adviser admits to contact with DNC hacker	The Hill	42,891	1089
38	4 April 2017	Russian Hackers Are Working To Amplify Donald Trump’s Wiretapping Claim, Expert Warns	HuffPost	47,481	1089
39	10 April 2017	Russian hacker arrested in Spain over ‘links to Trump victory’	The Local	50,898	3388
